# Who Is Responsible for a Dialogue Breakdown? An Error Recovery Strategy That Promotes Cooperative Intentions From Humans by Mutual Attribution of Responsibility in Human-Robot Dialogues

**DOI:** 10.3389/frobt.2019.00029

**Published:** 2019-04-24

**Authors:** Takahisa Uchida, Takashi Minato, Tora Koyama, Hiroshi Ishiguro

**Affiliations:** ^1^Advanced Telecommunications Research Institute International, Kyoto, Japan; ^2^Graduate School of Engineering Science, Osaka University, Osaka, Japan

**Keywords:** android, conversational robot, dialogue strategy, error recovery, dialogue breakdown

## Abstract

We propose a strategy with which conversational android robots can handle dialogue breakdowns. For smooth human-robot conversations, we must not only improve a robot's dialogue capability but also elicit cooperative intentions from users for avoiding and recovering from dialogue breakdowns. A cooperative intention can be encouraged if users recognize their own responsibility for breakdowns. If the robot always blames users, however, they will quickly become less cooperative and lose their motivation to continue a discussion. This paper hypothesizes that for smooth dialogues, the robot and the users must share the responsibility based on psychological reciprocity. In other words, the robot should alternately attribute the responsibility to itself and to the users. We proposed a dialogue strategy for recovering from dialogue breakdowns based on the hypothesis and experimentally verified it with an android. The experimental result shows that the proposed method made the participants aware of their share of the responsibility of the dialogue breakdowns without reducing their motivation, even though the number of dialogue breakdowns was not statistically reduced compared with a control condition. This suggests that the proposed method effectively elicited cooperative intentions from users during dialogues.

## Introduction

Recently, many conversational robots have been studied. To develop a robot that can make natural chat-like conversation is meaningful for various scenarios (e.g., communication support for seniors or children). However, dialogue systems, which are required by conversational robots, cannot always respond appropriately because of misrecognitions in automatic speech recognition (ASR) or the limitations of natural language processing. Such inappropriate responses create dialogue breakdowns, which are a critical obstacle in developing conversational robots.

Studies on avoiding dialogue breakdowns focus on improving natural language processing technologies, generating appropriate responses based on dialogue databases (Bessho et al., 2012), and detecting them (Higashinaka et al., 2016). Although these studies are necessary for conversational robots, it remains difficult to completely avoid dialogue breakdowns, inferred from the fact that even humans cannot completely avoid them (Komatani and Kawahara, 2000). In human-human conversations, people can recover from listening and misunderstanding failures based on the dialogue context and the situation. On the other hand, existing spoken dialogue systems have difficulty recovering the dialogue context once it starts to fail. Therefore, a method to handle dialogue breakdowns is essential for conversational robots.

## Materials and Methods

### Solution

In human-robot conversations, a dialogue breakdown is generally caused by the robot's insufficient natural language understanding, which requires sophisticated technologies of natural language processing (e.g., context or situation recognition) that remain immature. Therefore, the user's cooperation is critical to avoid and recover from dialogue breakdowns. But users usually blame the robot's inferior linguistic ability when a dialogue breaks down without exhibiting a cooperative intention. To solve this problem, this paper focuses on a dialogue strategy through which users share the responsibility for dialogue breakdowns with the robot.

Existing studies on spoken dialogue systems have mainly focused on improving the system's capability to avoid dialogue breakdowns. A conversation is based on mutual cooperation among conversation partners (Grice, 1989). Therefore, not only improving the robot's dialogue capability is required but cooperative intentions must also be elicited from users to recover from dialogue breakdowns. Users are expected to cooperate with the robot if they share responsibility for dialog breakdowns. If the robot always blames the users for dialogue breakdowns, they might become uncooperative and lose the motivation to continue talking. Therefore, the robot should help the users identify their own responsibility for dialogue breakdowns without extinguishing their motivation.

What kind of dialogue strategies lead users to accept responsibility without reducing motivation? When a dialogue breakdown occurs, the robot has three choices: it can blame itself, the user, or nobody. In this study, the robot follows psychological reciprocity when it apportions responsibility. Psychological reciprocity is a social rule that people repay what they get something from another person (Cialdini, 1989). In this case, the robot first blames itself for the dialogue breakdown by apologizing: “I'm sorry, I couldn't catch that” or “Repeat that, please.” Then the user will probably feel responsible in the next dialogue breakdown owing to psychological reciprocity. In other words, the robot can make the user voluntarily feel responsible for a dialogue breakdown. In this way, the robot alternately attributes the responsibility to itself and to the user to balance the location of responsibility. We hypothesize that this dialogue strategy can balance the responsibility for the dialogue breakdown while maintaining the user's motivation to continue the dialogue and eliciting cooperative intention from the robot.

On the other hand, even if the robot alternately blames the dialogue breakdown on the user and itself, the user will probably not cooperate if he/she does not feel responsible. This study incorporates the user's guilt for the dialogue failure because one function of guilt is to accept one's own responsibility (Lewis, 1971). The user's guilt can be elicited as getting angry or becoming irritated. Therefore, the robot should express such negative emotions to create guilt in the user when a dialogue fails.

If a dialogue breakdown occurs repeatedly even though the robot expresses frustration at the user, the user is probably not being sufficiently cooperative. In this case, the robot expresses stronger emotional expressions when the dialogue fails. A robot naturally gets angrier and angrier with more dialogue breakdowns just as people do in human-human conversations. The user may feel that it is strange and unnatural if the robot does not change its emotions despite repeated dialogue failures. Our proposed strategy is that at the beginning of a dialogue, the robot should alternately attribute the responsibility for the dialogue breakdown to itself and to the user and express stronger negative emotions if the dialogue breakdowns persist.

**Figure 1:**
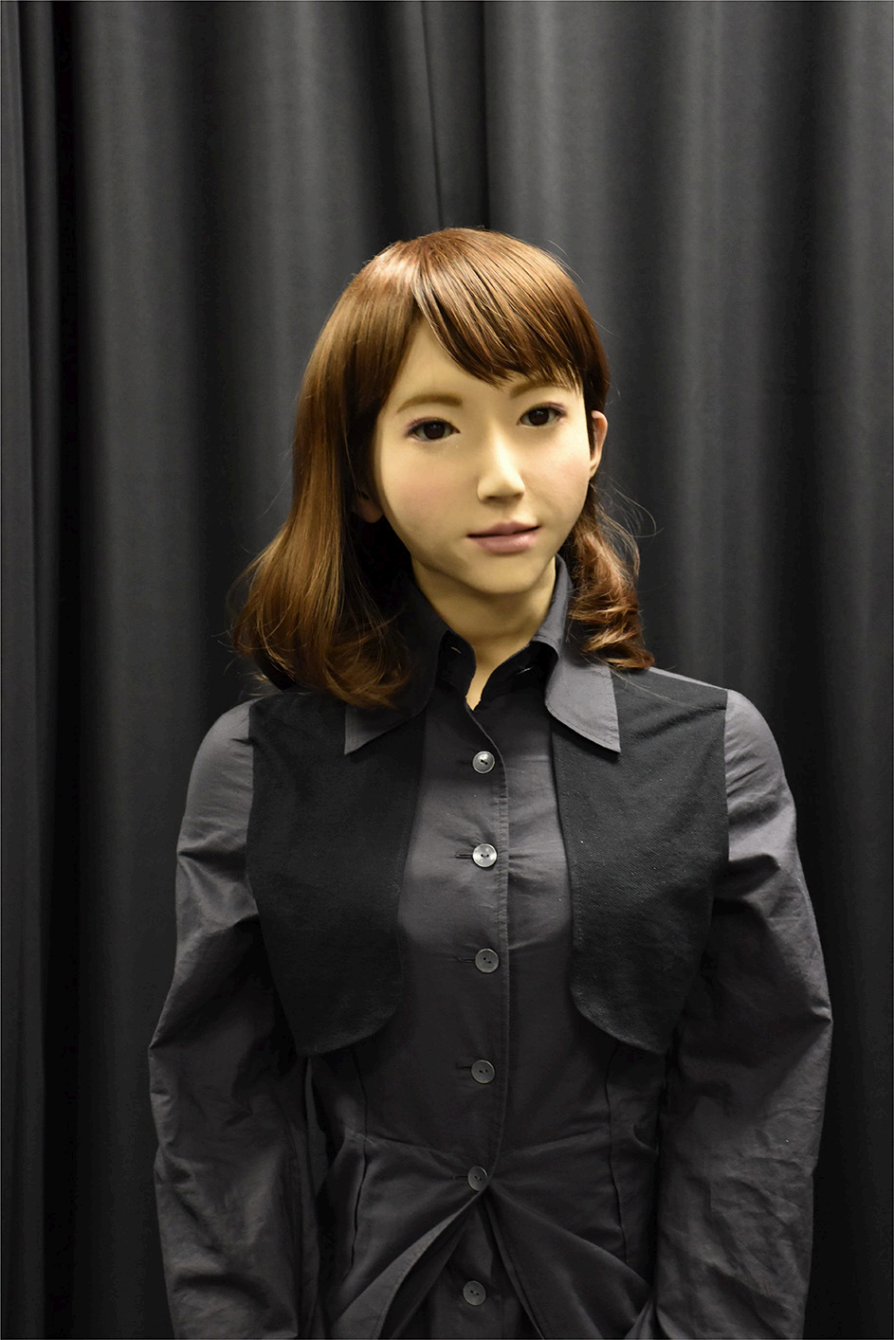
ERICA.

To test the above idea, a robot needs the ability to express rich emotional expressions. In this study, we use an android named ERICA that has many actuators in its face and a very human-like, feminine appearance (Figure 1). We implemented the dialogue strategy in it and experimentally verified its effectiveness.

### Related Work

Many studies have argued that psychological reciprocity is an essential factor in human-human conversation. Reciprocity is based on people's expectation that when an action committed by one individual positively affects another, an action is returned with an approximately equal positive effect (Burgoon et al., 1995). Studies on human-agent interaction report that people prefer a personal computer that praises them even though it is a non-human agent (Festinger, 1954). That is, psychological reciprocity occurs not only in conversations among people but also between people and artificial agents.

Some studies on reciprocity have focused on mutual self-disclosure in human-human conversations (e.g., Jourard, 1971). Also in human-robot interaction, the robot's self-disclosure behaviors encourage reciprocal self-disclosures from people toward it (Shiomi et al., 2015). However, no current dialogue systems apply the rule of reciprocity to the attribution of responsibility for dialogue breakdowns.

As described above, existing studies on dialogue systems have mainly focused on how to improve the robot's ability to avoid dialogue breakdowns. Some studies tackled how to recover from them. For example, Kitaoka et al. (2006) developed a system that effectively re-asks users when their recognition is inappropriate in a task-oriented dialogue. But the target of this paper is identifying what recovery strategies can maintain the user's motivation for dialogues in non-task oriented dialogues like casual conversations. Our study elicits cooperative intentions from users toward a robot by attributing the responsibility for dialogue breakdowns to users with the following rules of reciprocity.

### Error Recovery System

We developed an autonomous conversational android robot with the above dialogue strategy. This section describes our system's overview.

#### Errors in Dialogue

##### Listening error

Listening errors occur when ASR recognizes the user's speech without sufficient confidence. Since the system cannot respond because the recognized result is unreliable, the dialogue stops. Komatani and Kawahara (2000) proposed a system that decides whether to accept, check, or reject the recognized result based on confidence levels. Our system judges whether listening error happens based on the confidence measure (CM) scores of Julius ASR (Lee and Kawahara, 2009), a Japanese continuous speech recognizer.

##### Understanding error

Understanding error is equivalent to a case where people cannot understand what the interlocutor is talking about in a human-human conversation. Our system recognizes this error as follows. It creates a robot's utterance by following a dialogue scenario that is designed in advance. A statistical response method based on big databases (e.g., Bessho et al., 2012) is not used. Since such databases consist of the utterances of various people, it is difficult to maintain contextual consistency in the robot's utterances. For example, the robot may say, “I like apples,” even though it already said that it doesn't like them if its dialogue system refers to the utterances of a different person in the database. Such inconsistent utterances are obviously deleterious to user motivation in dialogues. Therefore, we designed a system that follows a dialogue script prepared in advance and generates responses based on matching keywords, which are determined by a script. When a user makes an utterance that does not include the keywords that are assumed to be in the script, the system judges that an understanding error has occurred.

##### Speech recognition error

This error, which is caused by voice recognition failure, resembles mishearing during human-human conversations. Here, even though the CM score is high, the recognized result is wrong. Consider a scene where the system asks, “can you drive?,” and the user answers “I can't drive.” The system might mistakenly recognize the user's utterance as “I can drive.” If the CM score is sufficiently high, it is difficult to judge the result as wrong since there is no contextual discrepancy. But a dialogue breakdown occurs if the system responds as follows: “let's go for a drive this weekend.” In this case, the user is expected to correct the system's response. Therefore, our system recognizes the speech recognition error from user feedback (negative words): “That's not what I mean! I can't drive.”

##### Interruption error

Interruption error occurs when the user suddenly introduces a subject outside of the context of the current dialogue. In human-human conversations, such abrupt topic changes sometimes occur when a speaker introduces a side sequence and inserts an irrelated topic in the main context (Jefferson, 1972). When we preliminarily tested our system on an android, some users interjected questions about its capabilities and features: “Can you see me?” and “how old are you?” Such error could also be regarded as understanding error, but our system judges it as interruption error and recovers in a different manner. Keywords about the android's capabilities and features are predefined, and the system recognizes errors when those keywords are detected in the user's utterance.

#### Dialogue Strategy to Promote Cooperative Intentions

In this paper, we propose a dialogue strategy that elicits the user's spontaneous responsibility in dialogue breakdowns. As mentioned above, the system is designed to promote a cooperative intention from users by having the robot blame either the user or itself to elicit responsibility or express emotion to induce guilt.

##### Alternate attribution of responsibility

When the system detects the above errors, it asks the user to repeat his question/comment and explicitly identifies who is responsible for the error: the robot or user. At that time, the attribution target for the responsibility is changed alternately between the robot and user as the number of errors increases. For example, when the robot blames itself for the error, it apologizes and explains: “I'm sorry, I didn't hear what you said, so please say it again.” By clearly apologizing, the robot takes responsibility for causing the error and encourages the user to speak more articulately so that it can easily understand. On the other hand, when the robot blames the user, it concretely states what the user needs to improve: “I didn't catch that, so please speak more clearly.” The robot first apologizes to elicits a cooperative intention; if the errors persist, it tries to understand what the user said by suggesting that he changes his manner of speaking. If the robot blames the user without first accepting its own responsibility, the user might feel uncomfortable and feel less motived to continue the dialogue. The robot must first accept the responsibility itself.

##### Increasing the degree of responsibility due to guilt

If dialogue errors continue even though the system adopts the above strategy, the user might not feel any responsibility. To increase the user's sense of responsibility for errors, the system makes him feel guilt for them. In this study, the robot adds an emotional content to the utterances when asking the user again because the number of errors is increasing. When accepting responsibility, it says: “Oh, I'm annoyed that I keep mishearing you. I'm sorry, could you repeat that again?” The robot sighs to display annoyance with itself and also expresses annoyance through language. By blaming itself, it appeals to the user's sympathy and induces feelings of guilt. On the other hand, when assigning responsibility to the user, the robot says: “What? I don't understand what you're saying.” In this case, it sighs to direct annoyance at the user and in language to show its feelings. It makes users feel guilt by directly blaming them and seeks their cooperation.

#### Flow of Error Recovery

##### Flow overview

This section describes the implementation of our error recovery procedure. An example of error recovery in a dialogue is shown below (translated into English from the original Japanese).

System: Well, recently many people are coming to see me. What do you do for a living?

User: I'm a pilot.

System: Pilot? What? Can you say that again? (Understanding error)

User: I fly airplanes.

System: Airplanes? What? (Understanding error)

User: That's right. My job is flying airplanes.

System: Well, you look young, are you a student?

User: Thank you. No, I'm not.

System: Really? Today, it seems hot.

User: Hmm …how …(something unclearly)

System: Eh? Can you say that again? (Listening error)

User: Hmm …(something unclearly)

System: I'm sorry. I didn't hear you. (Listening error)

User: I wonder if it's sunny today.

System: Well. I like cartoons. What's your favorite cartoon?

User: I don't like cartoons.

System: Really? You don't watch anime? Well, I'm from Tokyo, which prefecture are you from?

User: …(something unclearly)

System: I'm sorry, can you speak more loudly? (Listening error)

User: I'm from Mie.

System: It's easy to understand you when you speak clearly.

User: I'm sorry.

System: Are you from Mie?

User: Yes, I am.

System: Speaking of Mie, “Ise udon” is pretty famous there, isn't it?

User: How tall are you?

System: I've never been properly measured, but probably around 160 centimeters. What happened so suddenly? (Interruption error)

User: I thought you seemed tall.

**Figure 2:**
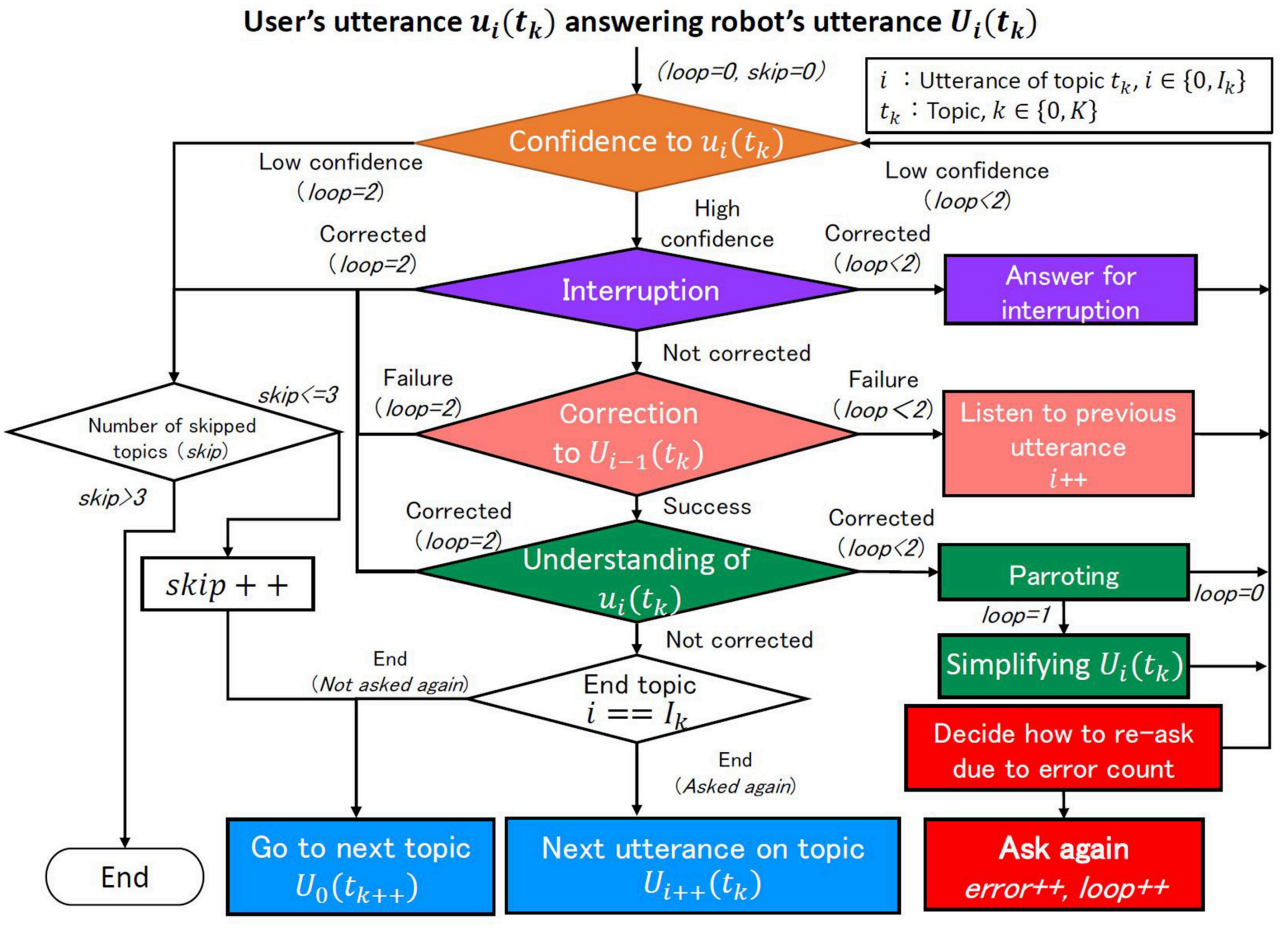
Flow of making robot's utterance.

Our system follows the strategy described in section Dialogue Strategy to Promote Cooperative Intentions and, as shown in the above example, changes the manner of inquiry depending on the error types described in section Errors in Dialogue: listening, speech recognition, interruption, and understanding. As shown in Figure 2, when ASR returns a result, the system checks the errors in the following order: listening, interruption, speech recognition, and understanding. Here, the issue is determining the priority of interruption error handling since there is some possibility that multiple types of error occur at the same time. We assume that the robot wants to avoid a situation where the user becomes less interested in the dialogue. If the robot ignores the user's utterances, the user's motivation will probably fall. Therefore, it handles interruption error with the highest priority and each error by the following process.

##### Handling listening errors

Let *u*_*i*_(*t*_*k*_) be the user's utterance recognized by ASR, which is a response to robot utterance *U*_*i*_(*t*_*k*_). Here *t*_*k*_ is the *k*-th topic in the dialogue (*k*∈[0, *K*]), where the topic is a coherent unit of such content as work or travel. *i* is the index of the robot's utterance in topic *t*_*k*_ (*i*∈[0, *I*_*k*_]). Since the dialogue in the topic follows a predetermined script, the index and the total number of utterances *I*_*k*_ are predetermined. When the system obtains user utterance *u*_*i*_(*t*_*k*_), it checks the CM score (*cmscore*∈[0, 1]) of the speech recognition. If the *cmscore* is lower than a predefined threshold, it judges that a listening error has occurred. The robot repeats the question and expresses emotion based the amount of error (*error*) that has occurred so far. This step can be repeated twice. If *cmscore* remains insufficiently high in the third re-inquiring, the system skips current topic *t*_*k*_ without replying and moves to the next topic:*t*_*k*+ 1_.

##### Handling interruption error

When the CM score exceeds the threshold, the system checks whether an interruption error (a sudden topic change) occurred. For example, the user suddenly asks about the robot's hobby when the topic was travel. In this case, the robot answers the interruption and rephrases its latest utterance *U*_*i*_(*t*_*k*_). The responses to the interruption are prepared in the script, and the system detects an interruption by keyword matching. If three or more interruptions occur to utterance *U*_*i*_(*t*_*k*_), then the system determines that continuing topic *t*_*k*_ is too difficult and abandons it.

##### Handling speech recognition error

When there is no interruption, the system checks for speech recognition error in the user's previous utterance *u*_*i*−1_(*t*_*k*_). If the system misrecognized an utterance, then perhaps robot's utterance *U*_*i*_(*t*_*k*_) was inappropriate and *u*_*i*_(*t*_*k*_) might be a correction candidate. The system recognizes the user's correction based on such negative keywords or phrases as “that's different” in such an utterance as “that's different from what I meant! I can't drive.” If the user corrected the robot's utterance, then the system addresses the speech recognition error. The robot returns to utterance *U*_*i*−1_(*t*_*k*_) with the same strategy as for the listening error. If an interruption occurs more than three times for utterance *U*_*i*_(*t*_*k*_), the system again judges that continuing topic *t*_*k*_ is too difficult and abandons it.

##### Handling understanding error

When the user doesn't correct her utterance, the system checks whether the keywords (assumed in the script) are included in user utterance *u*_*i*_(*t*_*k*_). If no keyword is found, the system recognizes that an understanding error has occurred. Then the robot follows the dialogue context that is defined in the script (i.e., skips the current topic and moves to the next one). However, the user's motivation for continuing the dialogue might decrease if the robot ignores the user's utterance and changes the topic. Therefore, the robot behaves as if it intentionally changed the dialogue topic after just partly understanding the user's utterance. When the system recognizes the understanding error, it first parrots words that it confidently recognized in the user's utterance to urge him to rephrase the last utterance. Such parroting is repeated several times, and if the understanding error continues, the system skips the current topic and starts a new one.

Here, perhaps the CM scores of some words in the user's utterance are sufficiently high even if the total confidence level in its entire sentence is low. In this case, a parroting strategy may fail if the robot pretends that it just partly understands the user's language. Therefore, the parroting strategy is only used when the confidence level of the whole sentence is high. In this paper, the robot parrots up to three parts of speech (noun, adjective, or verb) with a high *cmscore* whose threshold is empirically determined to be 0.6. At this time, verbs are converted to their basic form (infinitive). For example, when the robot asks, “What foreign country would you like to visit?,” and user might answer, “Unfortunately, I cannot afford overseas travel.” If the script does not assume such an answer, the robot parrots: “Afford?”

Parroting is repeated twice, and the robot moves to a new topic if the error remains. In the first parroting, the robot parrots some words and rephrases the robot's last utterance *U*_*i*_(*t*_*k*_); if the words have high *cmscore*s, it just rephrases them. In the second parroting, *U*_*i*_(*t*_*k*_) is simplified to narrow down the answer choices (e.g., a yes/no question or a two-choice question). In the case of the above example, if the user repeats the same response, after the parroting, the robot might continue the topic of overseas travel: “I heard that Singapore is a popular travel spot. Would you like to go there?” If the system cannot recover from the error, it skips current topic *t*_*k*_ and moves to the next topic: *t*_*k*+ 1_.

##### Other handling situations

If no error is detected, the robot's next utterance, *U*_*i*+1_(*t*_*k*_), is determined by the script. If the dialogue reaches the end of topic *t*_*k*_ (*i* = *I*_*k*_), the system goes to the next topic, *t*_*k*+1_, and also counts the topic skips (*skip*), which denote abandoning the attempt to understand the user's utterance. If the skip count (*skip*) exceeds three in the dialogue, the robot judges itself that this dialogue is unsuitable with this conversation partner and abandons it.

#### Autonomous Conversational Android Robot

We used an android named ERICA (Glas et al., 2016; Figure 1) whose appearance closely resembles a human female. It has a 12 controllable degrees of freedom for the eyebrows, eyelids, cheeks, lips, and the corner of the mouth and can express pleasure, anger, regret, and so on. It can look at a user whose location is measured by Microsoft's KINECT. We adopted Julius (Lee and Kawahara, 2009) for the ASR software and the VOICE TEXT ERICA of the HOYA Corporation (http://voicetext.jp) for the speech synthesis. When the android speaks, it moves its lips, head, and torso in synchronization with the prosodic features of its voice. Those movements are automatically generated based on an existing system developed by Ishi et al. (2012) and Sakai et al. (2016).

### Experiment

#### Aim

We implemented our error recovery system in ERICA and verified whether the android can make users feel responsible for dialogue errors without diminishing their motivation to continue the dialogue and talking cooperatively.

#### Experiment Conditions

Our experiment tested whether the proposed recovery strategy effectively elicits user feelings of responsibility for dialogue errors. The control conditions have two possible conditions: one is that the android always blames the user for the error, and the other is that it always takes responsibility for causing the error. Since users are more satisfied with a human-robot collaborative task when they and robot mutually adapt to each other (Nikolaidis et al., 2016), this paper avoids one-sided responsibility-attribution. Moreover, users reduce their motivation for continuing dialogue if the android always attributes the responsibility of dialogue breakdown to them. In addition, people feel less reliability, understandability, and trustworthiness for faulty robots (Salem et al., 2015). This suggests that the users were disappointed by the android and reduce their motivations if the android always accepts responsibility. Hence, in this experiment, we compared two conditions: a condition for the mutual attribution of responsibility for dialogue breakdown (experimental condition) and another that didn't assign blame to the conversation partner (control condition). In the control condition, we prepared the recovering behavior of several types of re-asking utterances without the attribution of the responsibility. The utterances in the experimental and control conditions are shown below.

Experimental condition

1. What? Can you say that again?
2. Huh? What's that?
3. I'm sorry. I didn't hear you.
4. I didn't understand that very well, I'm sorry, could you speak more clearly and plainly?
5. What? I didn't hear you. Can you speak more clearly?
6. What? Please speak louder and more plainly.
7. Ah… (annoyed with itself) I'm sorry, but can you repeat that?
8. Ugh… (annoyed with itself) Once again, thank you.
9. Aw… (blaming the user) So what?

Control condition

1. What? Can you say that again?
2. Huh? What's that?
3. What did you say?
4. What was that again?
5. Can you say that again?
6. Well, what?
7. Umm, what is it?
8. Would you repeat that again?
9. What did you just say?

In the experimental condition, phrases 1 and 2 are merely for re-asking. ERICA claims responsibility in phrases 3, 4, 7, and 8. Phrases 5, 6, and 9 are the opposite. The android expresses annoyance in phrases 7 and 8. Phrase 9 blames the user. In the control condition, all the phrases are merely for re-asking. In both conditions, the android changed the re-asking utterances in the order of the above list (that is, the *i*-th phrase is used in the recovery for the *i*-th error). If the recovery count exceeds nine, a phrase is randomly chosen from 8 or 9.

#### Procedure

**Figure 3:**
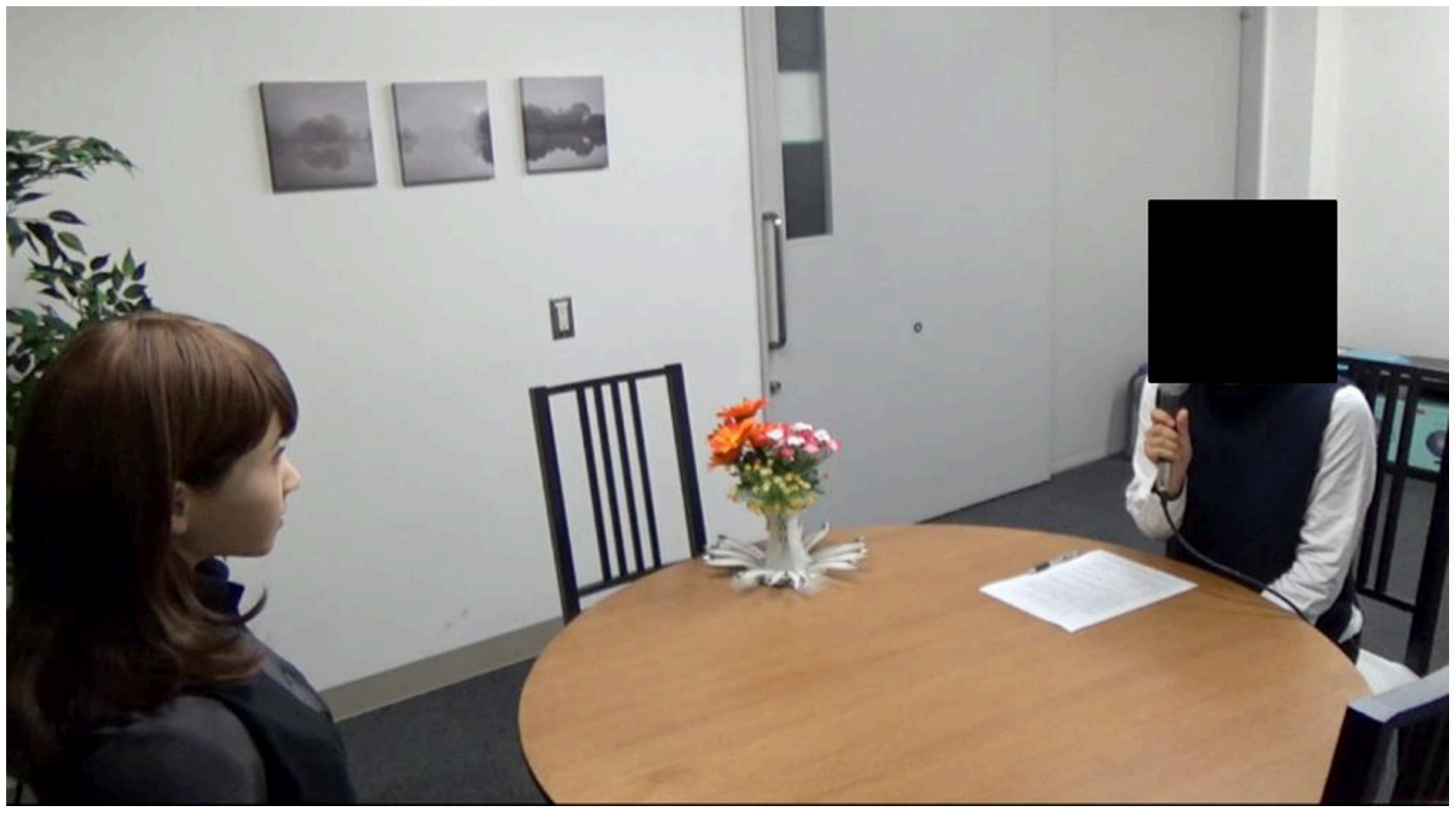
Experimental scene.

We compared the two conditions in a between-subject design to avoid the influence of repeated interactions of the same dialogue content. The participants interacted with ERICA, as shown in Figure 3. ERICA and the participants introduced themselves at the beginning of their dialogue and talked about the following 12 topics in this order: jobs, weather, cartoons, hobbies, hometown, travel, summer vacation, festivals, current events, artificial intelligence, automatic driving, and smartphones. They talked about each topic for several turns. The required time for the dialogue averaged about 10 min, although it varied depending on the number of errors.

**Figure 4:**
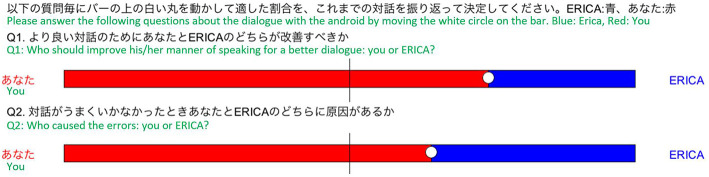
Questionnaire: responsibility attribution.

After a dialogue with ERICA, the participants answered a questionnaire about responsibility attribution and their impressions of ERICA, themselves, and the dialogue. We asked two questions about responsibility attribution: (Q1) “Who should improve his/her manner of speaking for a better dialogue: you or ERICA?” and (Q2) “Who caused the errors: you or ERICA?” The former addresses the participants' intentions to cooperate, and the latter addresses their sense of responsibility for the errors. For these questions, they were required to balance their contribution to the improvement and the cause between ERICA and themselves. We used an interface on a computer screen (Figure 4), where the length of the red bar indicates the ratio of the participants and the blue indicates the ERICA ratio. We used this format because the participants can intuitively evaluate the balance of responsibility between ERICA and themselves. They could change the ratio by moving the white circle on the bar using a mouse. The circle can be fixed in an analog scale.

The questions on their impressions of ERICA, themselves, and the dialogues are shown below. We used a seven-point Likert scale. The questions are described in the order of Q3, Q4, Q5, …, Q13, Q14, Q15.


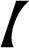
*Self-assessment of dialogue skills*
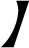
*Items for identifying bias of their own conversation skills between conditions*.

Q5: Do you think your speaking skills are good?

Q6: Do you think your listening skills are good?


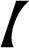
*Dialogue skill of ERICA*
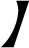
*Items for evaluating whether the impressions of ERICA's dialogue skill was changed by the proposed method*.

Q3: Do you think ERICA's skill of speaking is good?

Q4: Do you think ERICA's skill of listening is good?

Q12: Do you think ERICA was speaking naturally?


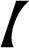
*Emotional expression of ERICA*
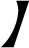
*Items for evaluating whether the participants actually felt ERICA's emotion in the proposed method*.

Q11: Do you think ERICA was actually irritated?


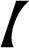
*Impression of ERICA*
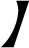
*Items for evaluating whether their impressions of ERICA were changed by the proposed method*.

Q7: Did you like ERICA?

Q9: Did you enjoy talking with ERICA?

Q10: Were you frustrated by ERICA?

Q13: Did you think you became closer to ERICA?


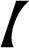
*Motivation to talk with ERICA*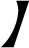 *Items for evaluating whether their motivation to talk with ERICA was changed by the proposed method*.

Q8: Do you want to talk with ERICA again?

Q14: Do you want to talk with ERICA more?

Q15: Do you want to talk with ERICA on another day?

## Result

**Figure 5:**
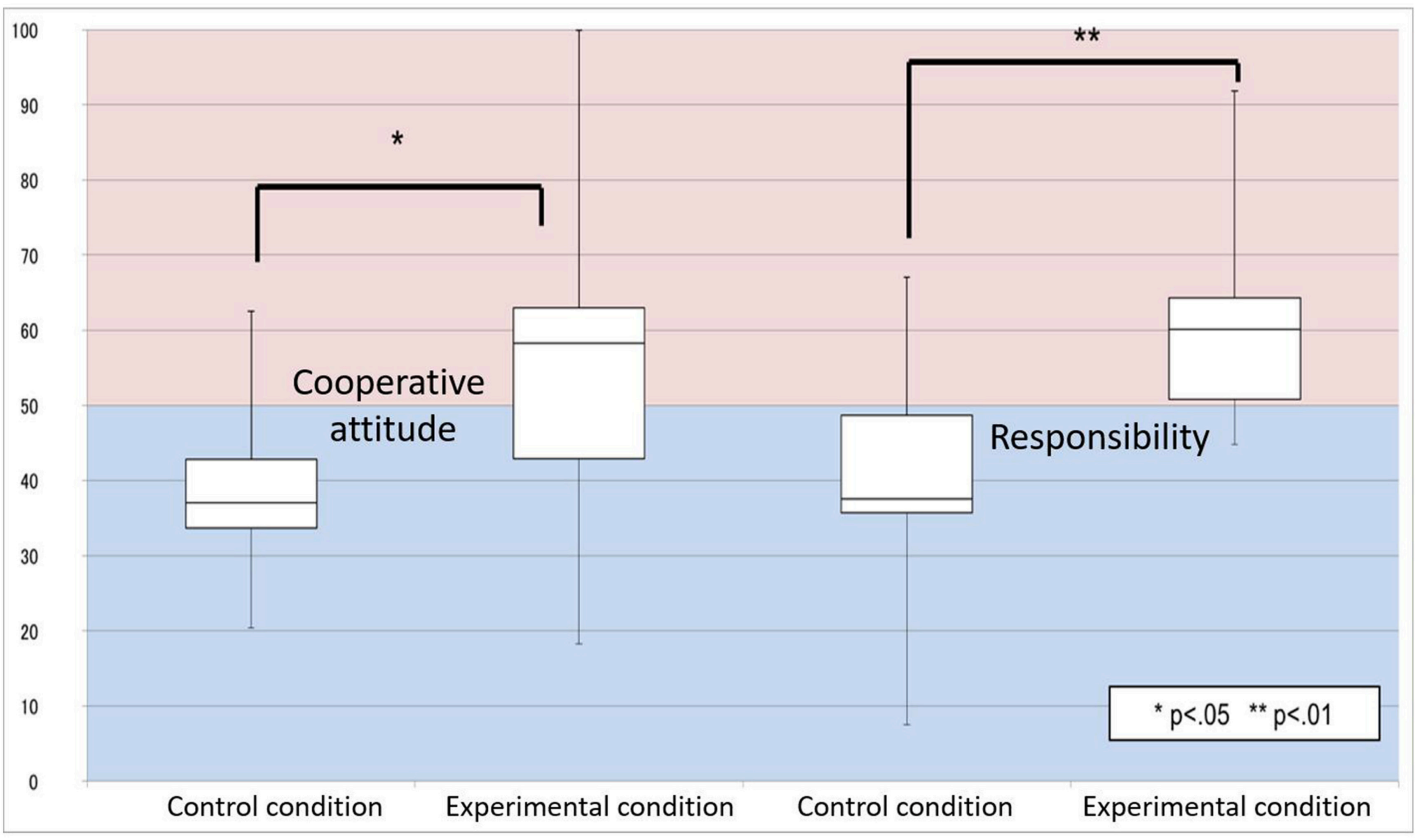
Result: responsibility attribution.

Fourteen Japanese people (seven males, seven females) participated in the experimental condition, and a different 14 (seven men, seven females) participated in the control condition. None of the participants had a history of neurological or psychiatric illness. All participants provided written informed consent prior to the start of the study, which was approved by the Ethics Committee of Osaka University, Japan. The questionnaire results of the responsibility attribution are shown in Figure 5. For both questions, a two-tailed *t*-test (we used a two-tailed *t*-test hereafter) confirmed a significant difference between the two conditions for cooperative intentions[*t*_(26)_ = 2.47; *p* = 0.02], and for responsibility [*t*_(26)_ = 3.80; *p* = 7.7 × 10^−4^]. These results suggest that in the dialogues, the participants attributed more of the cause of the dialogue errors to themselves based on the proposed strategy.

Table 1 shows the results of the impression scores. No significant differences were confirmed between the conditions of the self-assessment of dialogue skills (Q5, 6). Therefore, bias of the conversation skill between the conditions has less influence on the above results. We expected that ERICA's perceived conversation skill would be improved by the proposed method, but no significant difference was confirmed between the conditions (Q3, 4, and 12). The conversation basically followed the same script in both conditions: the contents of ERICA's speech and the timing of ERICA's listening were almost the same. This might have caused non-significant differences. In terms of ERICA's emotional expressions, the experimental condition is significantly higher than the control condition (Q11), confirming that the participants recognized that the android was annoyed at itself based on its utterances in the experimental condition. The participants' impressions of ERICA and their motivation to talk with ERICA were not significantly different among the conditions (Q7, 8, 9, 10, 13, 14, except for the score of Q15: “want to talk with ERICA on another day.” This suggests that the proposed method did not reduce their favorable impressions of ERICA even though she made irritating and blaming expressions toward the participants. The Q15 result suggests that the proposed method might promote motivation for long-term interaction without providing sufficient evidence to show that the motivation is promoted since there were no significant differences in Q8 and 14.

**Table 1:** Impression evaluation result.

**Item**	**Control condition**	**Experimental condition**	***t* (26)**	***p*-value**
	**Mean (SD)**	**Mean (SD)**		
Q5: Do you think your speaking skills are good?	3.93 (1.28)	3.29 (1.33)	1.25	0.22
Q6: Do you think your listening skills are good?	4.57 (1.55)	4.86 (1.12)	0.53	0.59
Q3: Do you think ERICA's skill of speaking is good?	5.07 (1.22)	5.86 (1.25)	1.62	0.12
Q4: Do you think ERICA's skill of listening is good?	3.07 (0.96)	3.79 (1.26)	1.62	0.12
Q12: Do you think ERICA was speaking naturally?	3.93 (1.39)	4.71 (1.48)	1.39	0.17
Q11: Do you think ERICA was actually irritated?	3.36 (1.49)	4.86 (1.30)	2.73	0.011
Q7: Did you like ERICA?	5.36 (0.61)	5.00 (1.13)	1.00	0.33
Q9: Did you enjoy talking with ERICA?	4.79 (1.26)	4.93 (1.49)	0.26	0.79
Q10: Were you frustrated by ERICA?	3.21 (1.32)	3.50 (1.18)	0.58	0.56
Q13: Did you think you became closer to ERICA?	4.36 (0.72)	4.07 (1.33)	0.68	0.50
Q8: Do you want to talk with ERICA again?	5.07 (0.70)	5.43 (1.18)	0.94	0.36
Q14: Do you want to talk with ERICA more?	4.93 (0.70)	5.43 (0.98)	1.5	0.15
Q15: Do you want to talk with ERICA on another day?	4.43 (1.40)	5.64 (1.17)	2.40	0.020

Finally, the amount of the android's re-asking in the conversation averaged 8.2 (*SD* = 3.07) in the control condition and 6.6 (*SD* = 4.58) in the experimental condition. A two-tailed *t*-test did not confirm a significant difference [*t*_(26)_ = 1.57; *p* = 0.13] between the two conditions. Therefore, the result failed to confirm that the participants spoke more clearly and in plainer language (that is, more cooperatively) in the experimental condition than in the control condition.

## Discussion

The experimental results show that the android successfully increased the participants' sense of responsibility for the dialogue errors through the proposed error recovery strategy. In addition, the participants in the experimental condition did not lose any motivation to talk with it, even though they felt that the android was more irritated at itself comparing with the control condition. These results suggest that the proposed method did not reduce the participants' motivation, even though it elicited a sense of their responsibility for the dialogue errors.

In the evaluation of the dialogue motivation, there is no significant difference in the two items: Q8 (“Do you want to talk with ERICA again?”) and Q14 (“Do you want to talk more with ERICA?”). Since the average scores of Q8 and Q14 are over 4 points (4 is neither yes or no) in both conditions, the motivation for the dialogues was not reduced by the proposed method. On the other hand, there is a significant difference in item Q15: “Do you want to talk with ERICA on another day?”. The first two items might mean that the participants wanted to talk with the android more deeply about the topics which they have talked about in the experiment. The last item is not limited to the topics, denoting motivation to talk with the android over a longer term. Therefore, the result suggests that the participants were motivated to engage in long-term dialogue without depending on topics under the proposed method. In the proposed method, since the responsibility for dialogue breakdown was attributed to each other, the android did not always have an intention to be superior or inferior to the participant. Consequently, the android with the proposed method might build more equal relationships with the participants and induce motivations for long-term dialogues. To confirm this idea, more detailed evaluations are required to investigate the relationship between users and the android.

On the other hand, we identified no significant reduction in dialogue errors due to the cooperative intentions of the participants. Although the proposed method evoked an intention to improve the dialogues, no behavioral changes (reducing the amount of dialogue breakdowns) were found during the ~10-min dialogues, probably because they were too short. A cooperative intention may reduce dialogue errors in longer dialogues with the android. In fact, when we look into the dialogue, some cooperative behaviors are observed. Below is an example of a dialogue between the android and a user in a laboratory demonstration (translated into English). In the beginning, the user's speech rate was 5.37 mora/s [User #1], but he spoke more slowly in the next four utterances [Users #2–#5] (4.06 mora/sec) after he was required to repeat himself. Moreover, he spoke more clearly and more plainly [User #7] after the second time the android re-asked [System #7] (he rephrased “a manufacturer of valves of fluid control devices” into “a valve manufacturer”). Even without the proposed strategy, users might adapt to the android's manner of speaking if they talk with it for a longer period. However, the proposed method is useful for them to quickly adapt to the android. If they need more time to adapt to conversational robots, their motivation for continuing the dialogue might be lost before they even begin to adapt. Adaptation time is critical for interaction with conversational robots. This example does not include utterances that attribute the responsibility to the user or utterances that induce guilt, because dialogue breakdowns are relatively rare. But we expect the proposed method to draw more cooperative behavior from the users since it elicits more cooperative intention than with the control condition. Future work needs to investigate how the proposed method alters user behaviors.

System #1: Oh, this is the first time to talk with you, isn't it?

User #1: Yes, it's the first time.

System #2: Um, I'm sorry. Can you say that again?

User #2: (muttering to himself: Should I speak in standard Japanese?) This is the first time.

System #3: I see. I'm sorry. Nice to meet you. Can I have your name?

User #3: I'm xxx.

System #4: xxx-san. Good afternoon.

User #4: Good afternoon.

System #5: I'm ERICA. Nice to meet you.

User #5: Nice to meet you, too.

System #6: Well, recently many people are coming to see me. What do you do for a living?

User #6: Well, fluid, a manufacturer of valves for fluid control devices.

System #7: Er, can you say that again?

User #7: A manufacture of valves.

System #8: Valves? What?

User #8: Valves.

System #9: Er, you look young, are you a student?

User #9: Thank you. Many people say so, but I'm not a student.

System #10: Really?

So that users share responsibility for the dialogue errors, the proposed method combined two strategies: reciprocal attribution of responsibility and a gradual increase in the degree of responsibility by expressing the android's emotions. To strengthen the user's sense of responsibility, the latter strategy seems sufficient. However, if the android blames the users whenever a dialogue error occurs, their dissatisfaction will undoubtedly increase. If it always admits its responsibility, the cooperation of users might be elicited due to their sympathy for it, but this method may not be comfortable. The proposed method must balance those strategies and improve human-robot conversation. But the optimal balance remains unknown. For good balance, a further experiment is necessary to reveal how the two strategies contribute to sharing responsibility.

As described in section Experiment Conditions, this experiment compared two conditions and skipped two other possible conditions: a condition in which the android always attributes the responsibility for the errors to the user and another where the android always accepts responsibility. The psychological studies described in section Experiment Conditions implied that these methods fail to elicit cooperative intention and motivation to talk. But comparing the proposed method with these methods might be helpful to investigate human behaviors in the error recovery.

This paper measured the number of times that the android had to re-ask to evaluate the participants' behavior changes, but more detailed behavior analysis would be helpful to understand the proposed method's effect. The changes in such speech behaviors as utterance length, the number of utterances, and speech rate are related to cooperative behaviors, and they should be analyzed in a future study. Since there is individual variation on these speech behaviors, analysis needs to take personality traits into consideration.

The proposed method is designed to elicit the affective states of the users' (guilt/shame) that makes them feel responsibility for dialogue errors. However, it is not clear that these affective states really changed and influenced their behaviors. We must scrutinize how the proposed method altered the participants' behaviors. Moreover, perhaps the system can be improved if the user's affective states are also fed back to the dialogue strategy, as suggested by Costa et al. (2018). The method proposed by Paquette et al. (2016) might be useful to estimate affective states in real-time. Such improvements are also future work.

The dialogue sequences were scripted and the script was pre-defined in the experiment. This was intended to prevent participants from engaging in dialogues with the android with unsuitable content. Previous work (Uchida et al., 2016) suggested that people do not receive information or understand the significance of the android's words when the dialogue content is unsuitable, that is, the android's opinion does not seem plausible. For example, people are not interested in the android's speech if it expresses a subjective opinion about the taste of a meal that it obviously cannot taste. In this study we designed the dialogue content so that it is appropriate for an android. However, this limits the method's generality with respect to dialogue content. Further research is required to explore the effect of more varied and unscripted conversations about dialog quality.

This study defined four types of dialogue error from the viewpoint of system implementation. This category is not necessarily optimal for designing a recovery strategy to elicit human cooperative behaviors and increase the dialogue's naturalness. Higashinaka et al. (2015) categorized the dialogue errors that people subjectively recognize. Comparison of the paper's error category with a subjective one would support analysis of the property of the errors to improve recovery strategy. That approach, which is outside the scope of this paper, is additional future work.

Concerning the above issue, the distribution of four types of dialogue errors depends on speech recognition, natural information processing, and the script management methods used in our system. It is useful for system improvement to know how the distribution changes when different methods are used. As in Xu's et al. (2009) study, evaluating the effectiveness of the proposed system is possible with a Wizard of Oz (WOZ) method. In other words, the effectiveness can be evaluated in the respect of error distribution when we artificially change the error distribution by WOZ. This is also future work.

Cultural differences have been reported in the manner of feeling guilty (Kluckhohn, 1960). Accordingly, cultural dependency might exist on the effectiveness of the proposed method. Furthermore, there are cultural differences in the impression of robots (Riek et al., 2010; Lee and Selma, 2014). In this experiment, we used an android since it can make rich emotional expressions. It is unclear whether this experiment's results can be applied to other dialogue robots with different capabilities of emotional expressions. Future work must clarify the scope of the applications of the proposed method by investigating cultural differences and robot types.

## Conclusion

This paper proposed a dialogue strategy to effectively elicit a cooperative intention from users for a conversational robot when dialogue breakdowns occur. Since these problems are mainly caused by the robot's insufficient natural language understanding, such user cooperation as speaking loudly and clearly in simple language is necessary to avoid and recover from them. Users are expected to be more cooperative when they share responsibility for these failures. However, their motivation for continuing a discussion will decrease if they are blamed by the robot. The proposed strategy solves this issue by balancing the responsibility between the users and the robot based on psychological reciprocity and expressing the robot's emotions to induce feelings of guilt. The experimental result showed that our proposed method caused the users to feel their own responsibility for the dialogue breakdowns without affecting their motivation. The method did not statistically reduce them in the experiment's short-term dialogues, although dialogue failures are expected to be avoided by a cooperative speaking manner by the users in long-term dialogues. A method that can elicit the cooperative intentions of users without losing their motivation will be useful technology in future human-robot conversations.

## Ethics Statement

None of the participants had a history of neurological or psychiatric illness. All participants provided written informed consent prior to the start of the study, which was approved by the Ethics Committee of Osaka University, Japan.

## Author Contributions

TU, TM, TK, and HI designed the research. TU, TM, and TK performed the research. TU and TM wrote this paper.

### Conflict of Interest Statement

The authors declare that the research was conducted in the absence of any commercial or financial relationships that could be construed as a potential conflict of interest.
